# Impaired retinal microcirculation in patients with Alzheimer’s disease

**DOI:** 10.1371/journal.pone.0192154

**Published:** 2018-02-02

**Authors:** Hong Jiang, Yi Liu, Yantao Wei, Yingying Shi, Clinton B. Wright, Xiaoyan Sun, Tatjana Rundek, Bernard S. Baumel, Jonathan Landman, Jianhua Wang

**Affiliations:** 1 Department of Ophthalmology, Bascom Palmer Eye Institute, University of Miami Miller School of Medicine, Miami, FL, United States of America; 2 Evelyn F. McKnight Brain Institute, Department of Neurology, University of Miami Miller School of Medicine, Miami, FL, United States of America; 3 Department of Ophthalmology, Third Affiliated Hospital of Nanjing University of Chinese Medicine, Nanjing, China; 4 Zhongshan Ophthalmic Centre, Sun Yat-sen University, Guangzhou, Guangdong, China; 5 National Institute of Neurological Disorders and Stroke, National Institutes of Health, Bethesda, MD, United States of America; Ehime University Graduate School of Medicine, JAPAN

## Abstract

The goal of this study was to determine the retinal blood flow rate (BFR) and blood flow velocity (BFV) of pre-capillary arterioles and post-capillary venules in patients with mild cognitive impairment (MCI) and Alzheimer’s disease (AD). Forty patients (20 AD and 20 MCI) and 21 cognitively normal (CN) controls with a similar age range (± 5 yrs) were recruited. A retinal function imager (RFI) was used to measure BFRs and BFVs of arterioles and venules in the macular region. The thickness of the ganglion cell-inner plexiform layer (GCIPL) was measured using Zeiss Cirrus optical coherence tomography. Macular BFRs in AD group were 2.64 ± 0.20 nl/s (mean ± standard deviation) in arterioles and 2.23 ± 0.19 nl/s in venules, which were significantly lower than in MCI and CN groups (P < 0.05). In addition, BFRs in MCI were lower than in CN in both arterioles and venules (P < 0.05). The BFV of the arterioles was 3.20 ± 1.07 mm/s in AD patients, which was significantly lower than in CN controls (3.91 ± 0.77 mm/s, P = 0.01). The thicknesses of GCIPL in patients with AD and MCI were significantly lower than in CN controls (P < 0.05). Neither BFV nor BFR in arterioles and venules was related to age, GCIPL thickness, mini mental state examination (MMSE) score and disease duration in patients with AD and MCI (P > 0.05). The lower BFR in both arterioles and venules in AD and MCI patients together with the loss of GCIPL were evident, indicating the impairment of the two components in the neurovascular-hemodynamic system, which may play a role in disease progression.

## Introduction

Adequate blood supply is critical to maintain normal brain function. Altered blood flow leads to neural dysfunction [1]. Cerebral hypoperfusion is evident not only in patients with Alzheimer’s disease (AD) but also in patients with mild cognitive impairment (MCI) determined by various imaging modalities [2]. However, whether the cerebral hypoperfusion is the cause or the consequence of neurodegeneration remains unknown, mainly due to the difficulty of direct visualization and assessment of the cerebral microvasculature and its link to cerebral neurodegeneration.

The retina and brain have the same embryological origin, and their microvasculature has similar anatomical and physiological features. Retinal vascular circulatory abnormalities could represent or mimic the cerebrovascular pathology. The retina’s neuronal and vascular changes are similar to changes known to occur in the brain. The retina is easily accessed by noninvasive optical imaging modalities and is thus readily studied [3,4]. The loss of retinal nerve fibers and neurons (i.e. ganglion cells), the thinning of the retinal nerve fiber layer (RNFL) and combined ganglion cell and inner plexiform layer (GCIPL) are detected by optical coherence tomography (OCT) and have been reported in patients with AD and MCI [5–8].

Another important component of the neurovascular-hemodynamic system is microcirculation. Decreased blood velocities in the retinal central veins were found in both MCI and AD, along with significant narrowing of central retinal venous column diameter in AD compared to MCI patients [4,9]. The alteration of microcirculation in the pre-capillary arterioles and post-capillary venules may be more sensitive in predicting the possible role of the vascular contributions on neurodegeneration. Imaging the microcirculation in the retina may also assist in establishing an easy access to inexpensive biomarkers of neurodegenerative disorders that could be used in evaluating treatment efficacy to prevent or slow the disease progression. The goal of this study was to determine the retinal microcirculation in patients with MCI and AD by measuring the blood flow rate (BFR) and flow velocity (BFV) in retinal arterioles and venules.

## Materials and methods

The study was approved by the institutional review board for human research at the University of Miami, and written informed consent was obtained from each subject. All subjects were treated in accordance with the tenets of the Declaration of Helsinki. AD and MCI patients were recruited from the McKnight Brain Aging Registry from October 2014 to December 2017. The patients were seen at the Division of Cognitive Disorders of the Department of Neurology at the University of Miami. The diagnoses of AD [10] and MCI [11] were made based on the National Institute on Aging-Alzheimer's Association (NIA-AA) criteria. A group consensus conference that included neurologists, psychiatrists, and neuropsychologists discussed and confirmed the diagnoses of these AD and MCI patients. CN controls were recruited during the same study period from subjects who received annual eye examinations or the family members of the patients. All enrolled participants were referred by recruiting clinicians participated in the study and no participants dropped out of the study.

A total of 40 patients (20 AD and 20 MCI) and 21 CN controls with a similar age range were recruited (Table 1). Patients with histories of other ocular or neurologic diseases that could affect the results, such as high refractive errors of more than +6.0 or -6.0 diopters, age related macular degeneration, diabetic retinopathy, glaucoma, cystic macular edema, cataracts and cornea disease, were excluded. Subjects with history of stroke, coagulopathy, uncontrolled hypertension and uncontrolled diabetes were also excluded.

**Table 1:** Demographics and clinical manifestations of patients and normal subjects.

	AD			MCI			Control			P Value
N	20			20			21			
Age	73.1	±	8.4	69.0	±	8.2	68.3	±	8.6	NS
Gender	10F, 10M			12F, 8M			11F, 10M			NS
SBP (mmHg)	136.4	±	18.4	129.2	±	16.6	136.8	±	19.0	NS
DBP (mmHg)	81.2	±	10.4	80.0	±	10.0	83.2	±	11.9	NS
MAP (mmHg)	99.6	±	10.7	96.4	±	11.5	101.1	±	12.1	NS
MPP (mmHg)	51.5	±	7.5	48.5	±	7.5				NS
HR (bpm)	68.2	±	15.5	66.5	±	11.5	64.0	±	9.7	NS
MMSE	22.4	±	4.8	27.7	±	1.4	29.5	±	0.7	P < 0.05*
Duration (yrs)	4.2	±	1.8	3.2	±	2.8				NS
Smoking	0			0			0			NS
Hypertension	11			9			12			NS
Dyslipidemia	14			11			10			NS
Diabetes	2			2			2			NS

Results are presented as the mean ± standard deviation. Abbreviations: AD: Alzheimer’s disease; DBP: diastolic blood pressure; DD: Disease Duration (years after onset of symptoms) CN, cognitive normal; HR: heart rate; MAP: mean arterial pressure; MPP: Mean perfusion pressure; MCI: Mild cognitive impairment; MMSE, Mini Mental State Examination; NS: not significant; SBP: systolic blood pressure; Hypertension: subjects with controlled hypertension; Dyslipidemia: subjects with controlled high blood cholesterol level on cholesterol medications; Diabetes: controlled type 2 diabetes without diabetic retinopathy; SD: Standard deviation

*P < 0.05 AD vs. control.

All participants had a screening ophthalmic examination by the investigator, including best corrected visual acuity, color vision, stereovision, intraocular pressure (IOP) measurements, a slit-lamp examination of posterior and anterior segment, and optical coherence tomography imaging (Cirrus, Carl Zeiss Meditec, Dublin, CA). Using the Cirrus OCT, a standard macular scan protocol using the 200 × 200 scan centered on the fovea was used to measure the average thickness of the ganglion cell-inner plexiform layer (GCIPL). The patients and CN controls underwent mini mental state examination (MMSE) by the PI or trained research associates. Patients and CN controls were asked to avoid large meals and not to ingest alcohol or alcohol-containing products before ophthalmic imaging. They were advised to avoid physical exercise for 24 h prior to the examination. Blood pressure and heart rate were measured. Mean arterial pressure (MAP) was calculated as 1/3 SBP + 2/3 DBP. The mean perfusion pressure (MPP) is determined by blood pressure and intraocular pressure [12]. MPP was calculated as 2/3[DBP + 1/3(SBP-DBP)]–IOP.

The RFI system (Optical Imaging Ltd., Rehovot, Israel) and its applications have been well-described in the literature [13–16]. RFI applies a stroboscopic light source and a high-resolution digital camera to rapidly take a series of retinal images. Using hemoglobin in red blood cells as the intrinsic motion contrast agent, the system measures the BFV of the red cell clusters in pre-capillary arterioles and post-capillary venules. No external contrast agent is needed, and the imaging procedure is non-invasive. The reproducibility of the measurements is 7.5%-11.0% [17]. RFI measures the blood flow velocities which are synchronized with the cardiac cycle to ensure the measurements at the same time point of the cardiac cycle.

**Fig 1:**
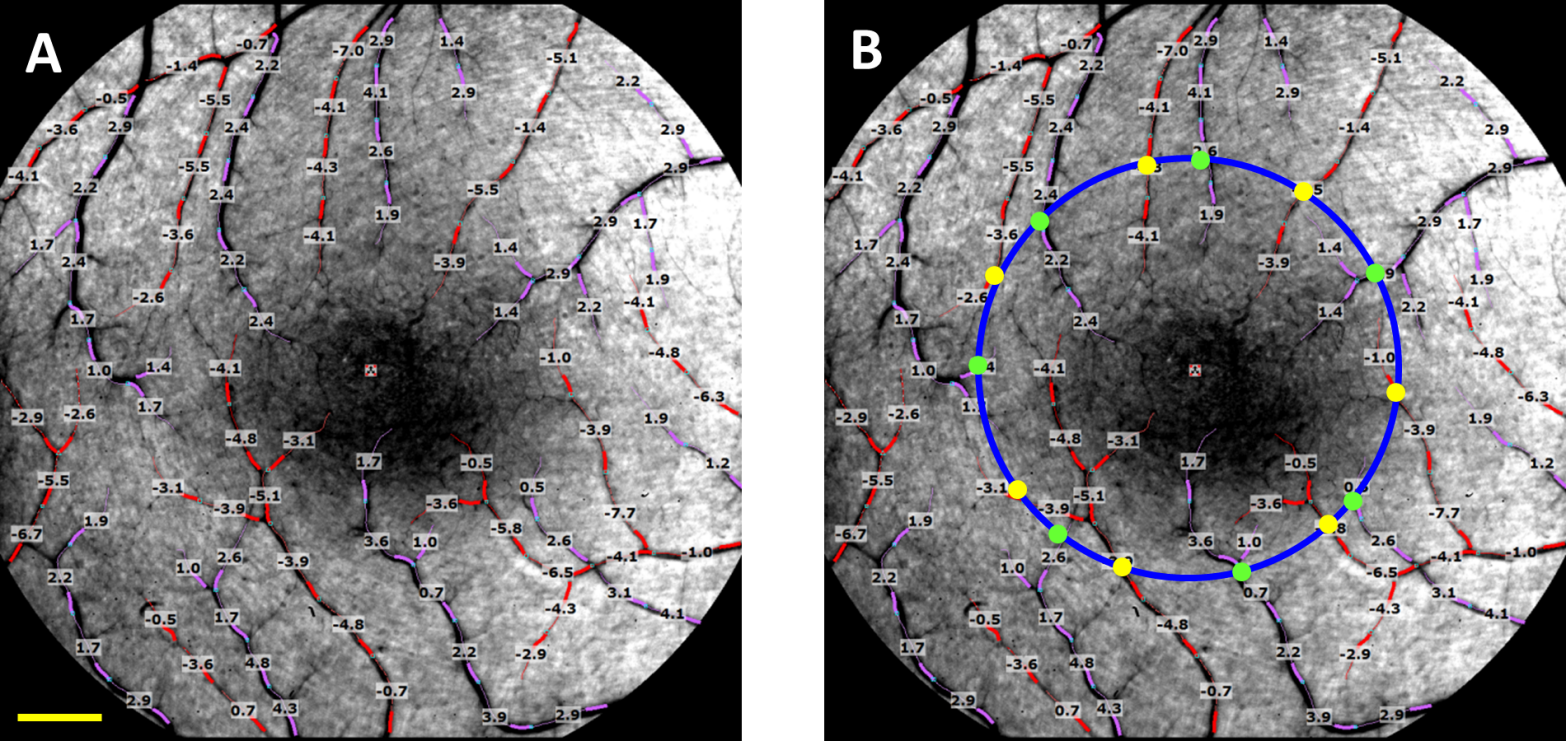
Quantitative retinal blood flow velocity map and macular blood flow. (A) The retina of a healthy subject imaged using the RFI device with a field of view of 20-degrees (4.3 × 4.3 mm^2^) centered on the fovea (dark area in the center) is shown. The arterioles are marked in red and overlaid with the measured blood flow velocities (mm/s); the venules and their respective velocities are marked in pink. The measured vessels cover the second, third and fourth branches of the retinal vessels. A negative value indicates blood flow away from the heart. In this case, the arteriolar flow moved towards to the fovea. A positive value indicates blood flow towards to the heart. In this case, the vessels are venules. (B) To analyze the macular blood flow, supplying the macular region including the vascular zone of the fovea, a 2.3 mm circle (blue) centered on the fovea was drawn and vessel diameters were measured in vessels at the locations (yell and green dots) crossing the circle. The diameter of the vessels which crossed the circle was determined. Blood flow of the vessel segment was calculated using the measured velocity and diameter for each arteriole (yellow dot) or venule (green dot). The total macular flow was the sum of all flow rates in the arterioles (all yellow dots) and venules (all green dots) which crossed the circle. The total macular flow rate of this healthy subject was 3.7 nl/s in the arterioles and 3.5 nl/s in the venules. Bar = 500 μm.

In the present study, each participant was asked to relax for 15 minutes in a semi-dark waiting room before imaging. The pupil was dilated with 1% tropicamide, and the macular region, centered on the fovea, was imaged with a field of view of 4.3 × 4.3 mm^2^ (setting 20 degrees) [3]. While the 20-degree field of view (FOV) has higher lateral resolution and shows the more visible tertiary branches of the retinal vessel, the image acquisition requires steady fixating and fine adjustment of the focus due to a relatively shallower focal depth. Alternatively, the 35-degrees FOV (7.3 × 7.3 mm^2^) has a deeper focal depth, which facilitates focusing on the retinal vessels. Both FOVs were used in clinical studies previously [3,18]. In the present study, the first choice of FOV setting was 20-degrees, and the second choice of FOV setting was 35-degrees. When the patients experienced difficulty in steadily fixating on the target, and sharp focusing was not achieved, the 35-degrees FOV was used. A conversion factor was used to convert the results from the 35-degree FOV to the 20-degree FOV for statistical analysis. The conversion factors of BFVs for the arterioles (f = 0.95) and venules (f = 0.92) were based on a previous study, which compared the results obtained from both fields of view [19]. More than four well-focused images from each image session and 3–5 overlapping sessions from each eye were obtained and used for calculating blood flow velocity. During the measurement, vessels were manually marked using the proprietary software (Browse, ver. 2.2.0.236) in the RFI. Once the vessels were marked, BFVs of the arterioles and venules were automatically calculated (Fig 1).

The macular region has an avascular zone (~0.5 mm in diameter) centered on the fovea, which is supplied by the terminal distributions of branches of the superior temporal and interior temporal arteries. The precapillary arterioles and postcapillary venules are staggered and pointing toward the center of fovea and the vascular layout of the inner retina provides an opportunity to quantify total blood flow to the macula [3]. Our prior work suggests that flow rates quantified in arterioles and venules crossing the parafoveal annulus provide equal and opposite values as would be expected by mass conservation [3]. To analyze the macular BFR supplying the macular region including the vascular zone of the fovea, a 2.3 mm circle centered on the fovea was drawn and vessel diameters were measured in vessels crossing the circle (Fig 1). The diameter of the vessels which crossed the circle was determined by counting the pixels of the full width and half of the maximum in the intensity profile, which was perpendicular to the vessel using custom software developed in Matlab (Mathworks Inc, Natick, MA) [3]. Blood flow of the vessel segment was calculated using the measured velocity and diameter using an equation [1/4 × π × (diameter)^2^ × velocity] [20]. The total macular flow was the sum of all flow rates in the arterioles and venules which crossed the circle.

A statistical software package (STATISTICA, StatSoft, Inc., Tulsa, OK) was used for descriptive statistics and data analysis. One-way analysis of variance (ANOVA) was used to analyze FBVs among groups. Post hoc tests with Fisher’s least significant difference (LSD) were used to test the pair-wise difference. Spearman rank-order correlation was used to evaluate the relationship among the parameters which were not normally distributed. The Spearman’s correlation coefficient (ρ) was reported. Chi-Square test was used to test these categorical variables including gender and the confounding factors. A result of P *<* 0.05 was considered significantly different. In addition, the sample size was calculated using a software program (Gpowr, ver. 3.0) developed by Erdfelder et al. [21]. The decrease of BFV of 18.5% was found in multiple sclerosis [3], and the repeatability was reported to be 7.5–11% [17]. To detect 15% of the change, a sample size of 13 subjects in each group would be enough to detect the true difference, with a detection power of 0.95.

## Results

**Fig 2:**
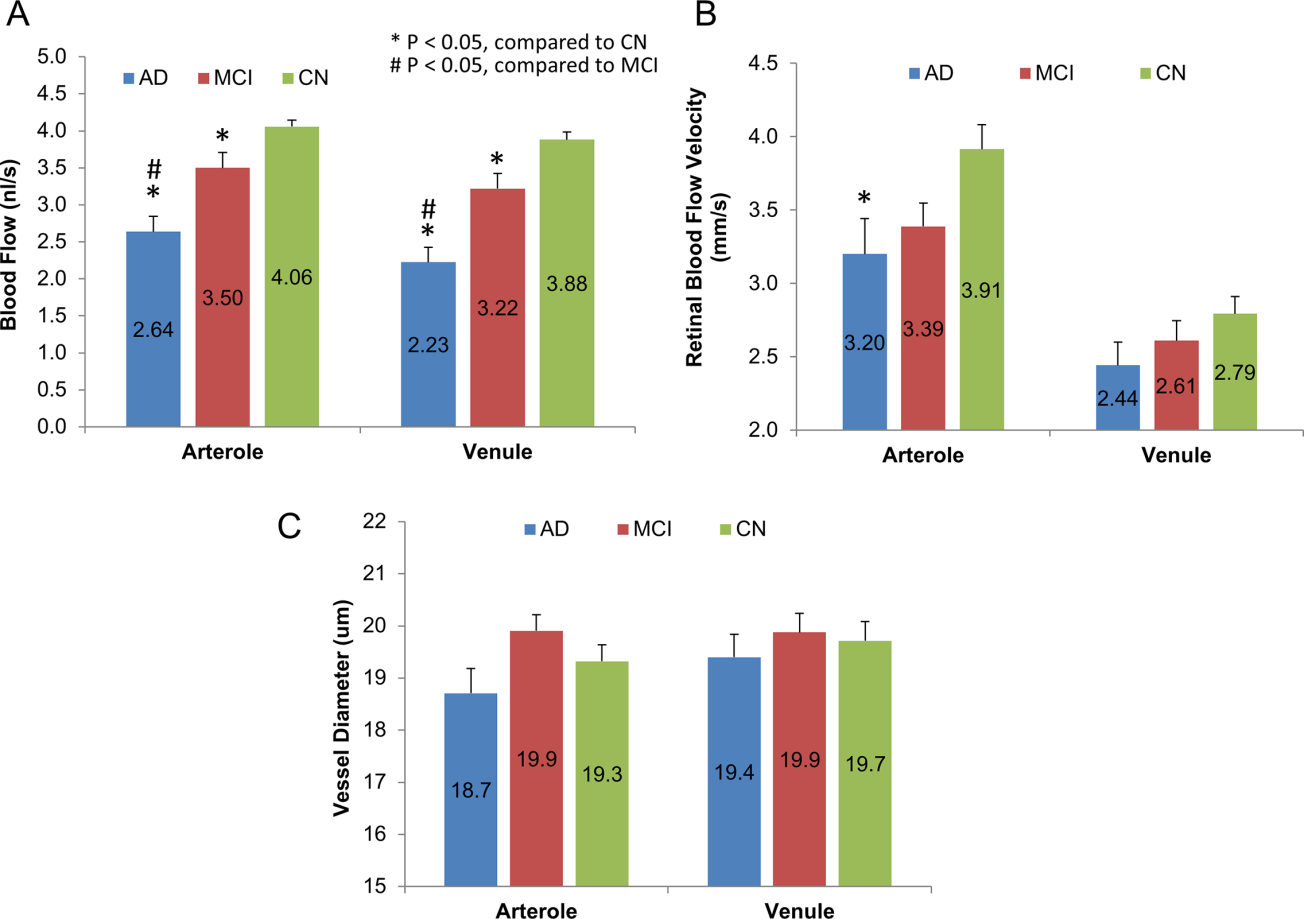
Retinal blood flow, velocity and vessel diameter in AD and MCI patients compared with CN controls. (A) Total arteriolar and venular blood flow rates were calculated in the macula. Macular blood flow rates in AD group were significantly lower than MCI and CN groups (P < 0.05). In addition, the macular blood flow rates in MCI were lower than CN in both arterioles and venules (P < 0.05). (B) BFVs of all arterioles and venules in the field of view were measured. The BFV in AD group was significantly lower than in controls in the arterioles (P = 0.01) but not in venules (P > 0.05). There were no significant differences of BFVs in arterioles and venules between AD and MCI (P > 0.05). (C) Vessel diameters were measured in the vessel segments crossing a circle (diameter 2.3 mm centered on the fovea) and there were no significant differences in both arterioles and venules among the three groups. Bars = standard error.

The baseline characteristics of patients are listed in Table 1. There were no significant differences in age, gender, heart rate, blood pressure and mean perfusion pressure (P > 0.05) among groups. Macular BFRs in AD group were 2.64 ± 0.20 nl/s (mean ± standard deviation) in arterioles and 2.23 ± 0.19 nl/s in venules, which were significantly lower than in MCI and CN groups (Fig 2, P < 0.05). In addition, BFRs in MCI were lower than in CN in both arterioles and venules (P < 0.05). There were no significant differences of BFRs between arterioles and venules in all three groups (P > 0.05).

The BFVs of the arterioles were 3.20 ± 1.07 mm/s in AD patients and 3.39 ± 0.70 mm/s in MCI patients. BFV of arterioles in AD was significantly lower than in controls (3.91 ± 0.77 mm/s, P = 0.01, Fig 2), while BFV of arterioles of MCI did not reach a significant level (P = 0.06) compared to CN controls. The BFVs of the venules were 2.44 ± 0.71 mm/s in AD patients and 2.61 ± 0.61 mm/s in MCI patients, which were not significantly different compared to controls (2.79 ± 0.54 mm/s, P > 0.05, Fig 2). There were no significant differences (P > 0.05) of BFVs in arterioles and venules between AD and MCI. There were no significant differences of vessel diameters of both arterioles and venules crossing the 2.3 mm circle among the three groups (Fig 2, P > 0.05).

**Fig 3:**
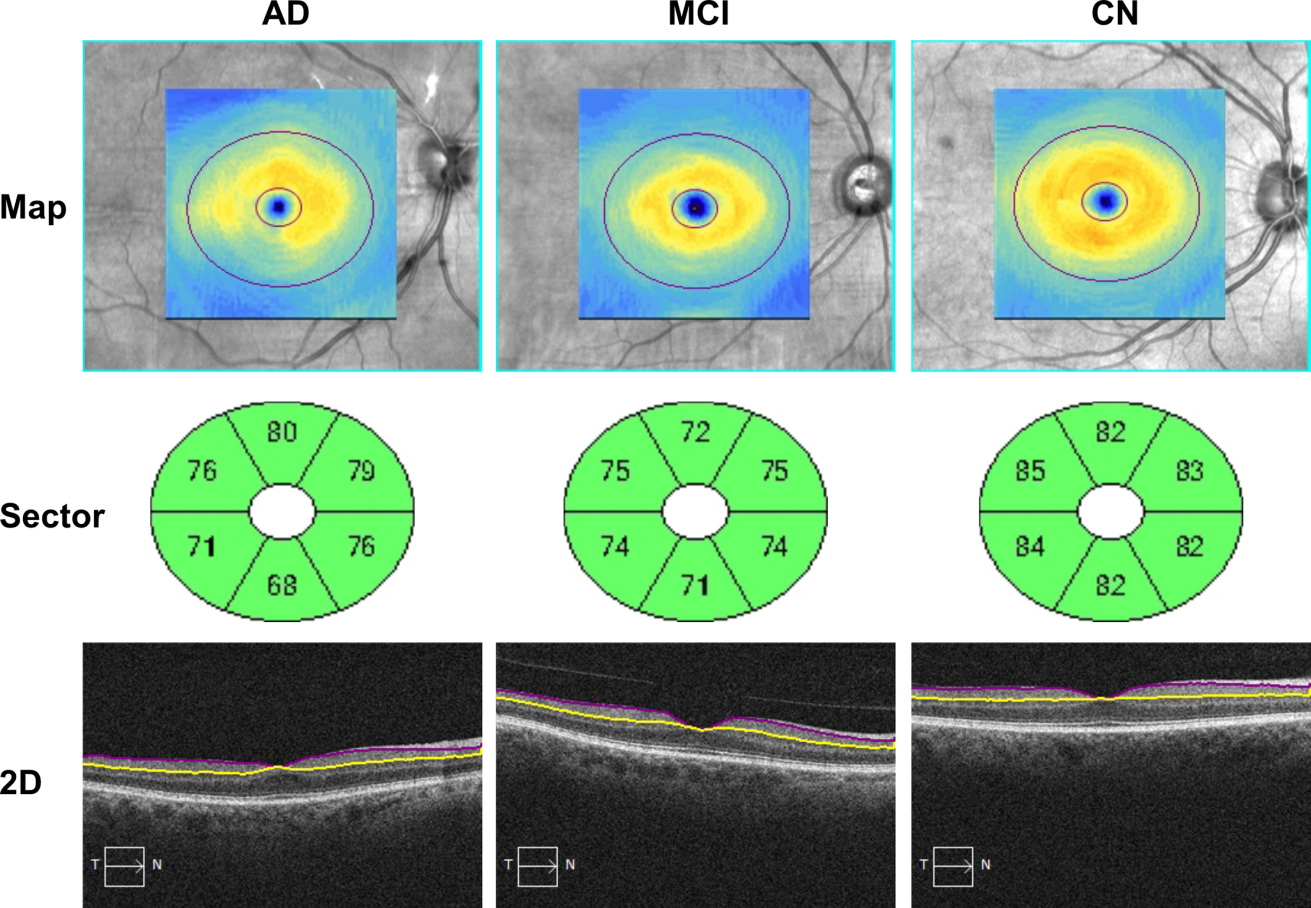
Representative thickness maps, sectoral thicknesses and two dimensional retinal images of ganglion cell-inner plexiform layer. The retina was imaged using Zeiss Cirrus optical coherence tomography. AD: Alzheimer’s disease; MCI: Mild cognitive impairment; CN, cognitive normal.

**Fig 4:**
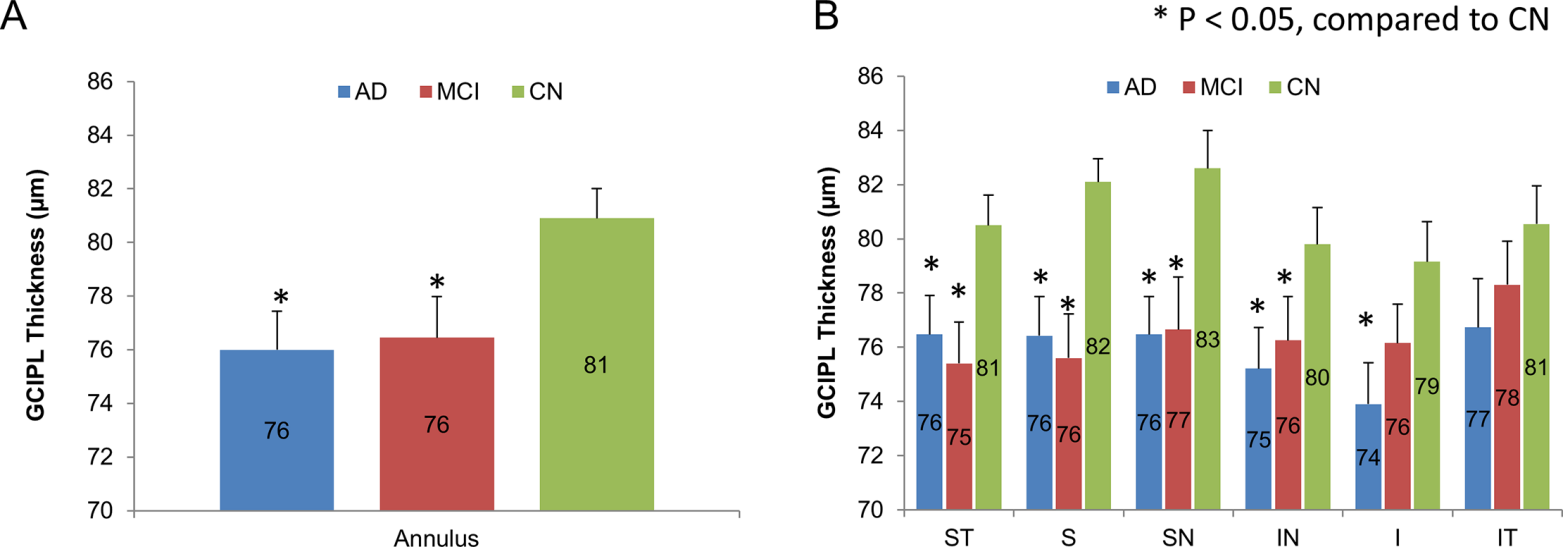
The thickness of GCIPL. (A) The thicknesses of GCIPL in the annulus of AD and MCI groups were significantly lower than the corresponding regions in CN group (P < 0.05). (B) The thicknesses of GCIPL in the superior temporal (ST), superior (S), superior nasal (SN), inferior nasal (IN) sectors of AD and MCI groups were significantly lower than the corresponding regions in CN group (P < 0.05). In the inferior nasal (IN) and inferior (I) sectors, GCIPL thickness in AD was significantly lower than CN (P < 0.05). No significant differences of GCIPL thickness were found in the inferior temporal (IT) among groups (P < 0.05). In addition, there was no significant difference of the thickness of GCIPL in the annulus and sectors between AD and MCI (P > 0.05). Bars = standard error.

The thicknesses of GCIPL in the annulus and superior temporal (ST), superior (S), superior nasal (SN), inferior nasal (IN) sectors of AD and MCI groups were significantly lower than the corresponding regions in CN group (P < 0.05, Figs 3 and 4). In the inferior nasal (IN) and inferior (I) sectors, GCIPL thickness in AD was significantly lower than in CN (P < 0.05). No significant differences of GCIPL thickness were found in the inferior temporal (IT) among groups (P < 0.05). In addition, there was no significant difference of the thickness of GCIPL in the annulus and sectors between AD and MCI (P > 0.05).

**Fig 5:**
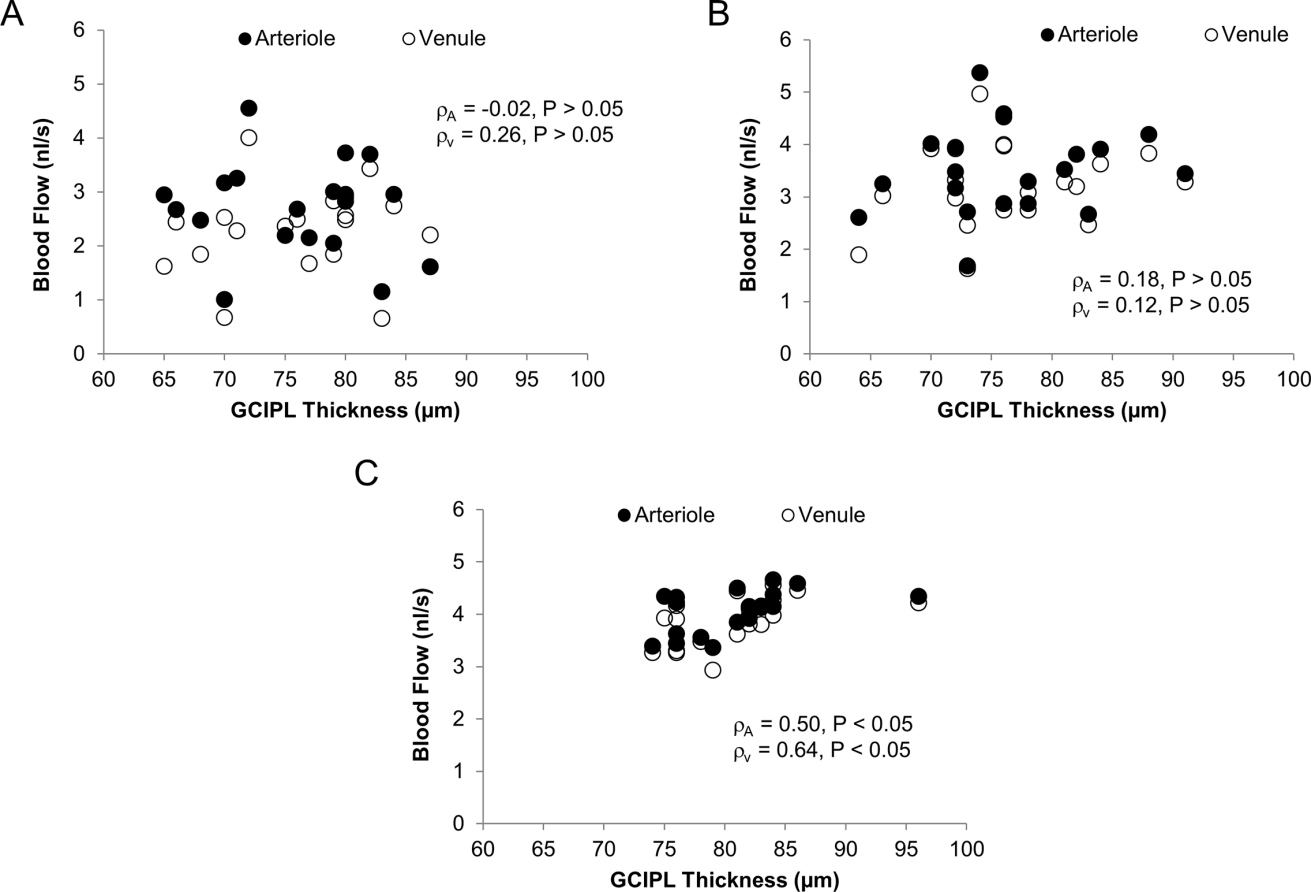
Relations between GCIPL thickness and macular blood flow. Annular GCIPL thickness and macular blood flow in both arterioles and venules were analyzed. There were no significant relations between GCIPL and blood flow rates in AD (A) and MCI (B). In contrast, GCIPL thickness was possibly related to blood flow rates in both arterioles and venules (C). Note both blood flow and GCIPL thickness spread out across the scales in AD and MCI groups, compared to CN group.

The BFVs and BFRs in arterioles and venules were not related to age, thickness of GCIPL (Fig 5), MMSE and disease duration in both AD and MCI groups (P > 0.05). In contrast, BFRs in arterioles and venules were positively related to annular GCIPL thickness in CN group (ρ = 0.50 for arteriolar BFR, ρ = 0.64 for venular BFR, P < 0.05, Fig 5). Arteriolar BFV was significantly related to diastolic blood pressure (DBP) in AD (ρ = 0.46, P < 0.05) and CN (ρ = 0.70, P < 0.05). In addition, arteriolar BFV was significantly related to mean arterial pressure (MAP) in AD (ρ = 0.53, P < 0.05) and CN (ρ = 0.63, P < 0.05).

When each segment of the vessels was counted, vessel diameters in both arterioles and venules were not related to BFVs in both arterioles and venules, but were positively related to BFRs in both arterioles and venules in each of the three groups (ρ ranged from 0.46 to 0.87, P < 0.05). BFVs in the arterioles and venules were positively related to BFRs in both arterioles and venules in each of the three groups (ρ ranged from 0.31 to 0.85, P < 0.05).

## Discussion

Our study showed that patients with AD and MCI had decreased macular BFR, which is in agreement with cerebral hypoperfusion previously measured in AD [22,22] and MCI [23]. Cerebral neurovascular homeostasis relies on the adequate cerebral blood flow to fulfill the cerebral metabolic demand [24]. In animal models, cerebral chronic hypoperfusion induces mitochondrial energy failure and oxidative stress resulting in neuronal death [25]. The findings in the eye in the present study may echo the vascular changes in the brain. It is reported that cerebral amyloid angiopathy characterized by the accumulation of Amyloid beta (Aβ) peptide in the tunica media and adventitia of the vessels caused vascular smooth muscle cell degeneration and loss of vessel wall integrity [26]. Cerebral amyloidal angiopathy was found to be one of the major pathologic changes in AD in addition to amyloid plaques and neurofibrillary tangles containing hyperphosphorylated tau [27,28]. The direct damage of the arterioles by Aβ deposition in the walls has been well documented in AD [29–31], which occurs in parallel with the formation of Aβ deposition in the brain parenchyma [32]. Furthermore, collagen fibril deposition in the walls of capillaries [33] and veins [34], resulting in narrowed or even luminal occlusion could contribute to the increased vascular resistance. The vasculature in the brain and retina shares similar anatomic and physiologic features. The amyloid β deposit was also seen in the retina [35,36], which may contribute to RNFL thinning, retinal central vein narrowing and decreased venous blood speed [9,36].

Although BFRs in the venules were decreased in both AD and MCI, decreased post-capillary venular velocities did not reach a significant level in patients with AD and MCI. This is not in agreement with a previous report that showed the decreased venous blood velocities of the central retinal venule in both AD and MCI patients compared to CN controls [4]. In the present study, the blood flow velocities in the smaller vessels (i.e., arterioles and venules) were measured. It is possible that the large veins may show the changes as the total blood flow is decreased, while the small venules may not show the detectable magnitude of the changes. Alternatively, the discrepancy may be due to the different cohort of patients and different imaging modalities.

The decreased BFV measured in the entire field of view was evident in AD, which is different from the effect of normal aging on retinal blood flow velocity. The age related decline of retinal venular velocity was reported, whereas the arteriolar velocity was not reported to be affected by aging [17,37]. The decreased pre-capillary arteriolar BFV may reflect the structural changes in the retinal microvasculature, manifesting in a sparser retinal vascular network showed on fundus photos from the large scale epidemiologic studies [38,39]. Interestingly, arteriolar BFV was found to be positively related with diastolic blood pressure (DBP) and mean arterial pressure (MAP) in AD. In the frail elderly, a value of DBP ≤ 60 mmHg was reported to be associated with reduced survival due to cardiovascular problems [40]. The blood pressure measurements (DBP, SBP and MAP) in the AD group in the present study were the same in CN controls. The positive correlation between DBP/MAP and arteriolar BFV may indicate a degree of compensation or regulation to maintain the constant perfusion while the arteriolar velocity in the retina may be low. However, this speculation needs validation in longitudinal studies with large sample sizes.

The thinning of GCIPL in both AD and MCI found in the present study supports previous findings [8,41]. The correlation between the thinning of GCIPL (cell bodies and dendrites) and the atrophy of gray matter volume of the occipital and temporal lobes in aging was reported [42]. In the present study, the decreased arteriolar BFVs and decreased blood flow rates in both arterioles and venules in AD patients coexist with the thinning of GCIPL. Similarly, the decreased blood flow in MCI patients coexist with the thinning of GCIPL. The structural and functional integrity of the nervous system relies on the homeostasis of the microenvironment, the balance between energy demand for neural activity, and nutritional support from the blood supply. This finding indicates that the neurodegeneration and the alterations of microcirculation of the retinal neurovascular-hemodynamic system coexisted in patients with AD and MCI. However, it is worth noting that the changes may not be parallel as the relation between vascular measurements and GCIPL thickness was not established in AD and MCI. While the blood flow and velocity showed a decreasing trend from CN to MCI and AD, no such trend of GCIPL thinning was evident in the present study. While this study could not be used to interpret whether impaired microcirculation is the cause or the consequence of neurodegeneration, it could be speculated that the impairment of the microcirculation may at least accelerate disease progression, which may facilitate the conversion of MCI to AD.

There are some limitations to the present study. First, the temporal relationships between the retinal microvasculature and neurodegeneration cannot be determined due to the cross-sectional nature of the present study. Second, the alterations of cerebral perfusion in patients with AD and MCI were not measured simultaneously, which prevented the comparison between the ocular and cerebral findings. Future studies with cerebral perfusion may address the relations between retinal and cerebral circulation. Third, not all patients and CN controls’ blood flow velocity were measured using the same settings of the field of view, which may induce some measurement errors. In a previous study [19], the difference between these BFVs using two different FOV was approximately 8%, which is much smaller than the difference (~16%) between the AD and CN groups in the present study. Therefore, the difference of the arteriolar BFV found in the present study most likely reflects the true difference. Finally, the vessel diameters were only measured in the vessels crossing the 2.3 mm circle for the purpose of blood flow calculation. These measured vessels were 3^rd^ branches of the arterioles and venules with a narrow range of vessel diameters. This may explain the reason why a relation between vessel diameter and velocity was not established in the present study. Further development to measure the vessel diameters of all vessels will provide more information on this relation.

In conclusion, we report the decreased blood flow in both arterioles and venules in AD and MCI patients together with the loss of GCIPL, indicating the alterations in the neurovascular-hemodynamic system, which may play an important role in disease progression.
